# Motor Skill Development at Preschool Age in Girls and Boys: The Role of Outdoor Free Play

**DOI:** 10.3390/children12050594

**Published:** 2025-05-02

**Authors:** Valentina Biino, Caterina Pesce, Clarice Martins

**Affiliations:** 1 Department of Neurosciences, Biomedicine and Movement Sciences, University of Verona, 37131 Verona, Italy; valentina.biino@univr.it; 2 Department of Movement, Human and Health Sciences, University of Rome ‘Foro Italico’, 00135 Rome, Italy; 3 Research Centre in Physical Activity, Health and Leisure and Laboratory for Integrative and Translational Research in Population Health, University of Porto, 4200-450 Porto, Portugal; clarice@fade.up.pt

**Keywords:** motor competence, spontaneous play, early childhood, sex difference

## Abstract

Background/Objectives: Trajectories of fundamental movement skill (FMS) development start diverging in females and males in early childhood, with determinants of this divergence spanning from individual to social and environmental factors. The present cross-sectional study focuses on the role of free outdoor play and aims to investigate whether sex differences in FMS typically observed in early childhood are associated with participation in free outdoor play. Methods: One hundred and forty-two children aged 4.3 ± 0.8 yrs were evaluated for locomotor and object control skills (TGMD-3), weight status (BMI), and free outdoor play (parent-reported). Motor skill competence scores were submitted to moderated regression analyses to evaluate the individual and joint effects of sex, outdoor play, age, and BMI; interrelations among these variables were also estimated with network analysis. Results: Results of the moderated regression showed, beyond the expected prediction of motor skill competence (overall and object control skills) by sex and age, also a significant sex x outdoor play interaction, with higher motor skills being predicted by more frequent outdoor play in males only. The network analysis confirmed a positive association between outdoor play and motor skill competence in males but not in females. Conclusions: Males might capitalize on free outdoor play opportunities as early as preschool age to engage in activities that promote their motor and especially object control skill development. Longitudinal studies are needed to test causality and derive practical indications for enabling both males and females to fully exploit the opportunities provided by free outdoor play to exercise both locomotor and object control skills.

## 1. Introduction

The accelerated development of the nervous system during infancy and early childhood, and the close interrelation of the development of the neural substrates involved in the control of movements and the goal-oriented planning and execution of movement actions [1,2] make the early years a critical period for the development of fundamental motor skills (FMS). FMS are basic gross motor skills, categorized into locomotor (e.g., running, jumping), object control (e.g., throwing, catching), and stability skills (e.g., balancing, bending) that serve as the foundational building blocks for physical activity and sport [3,4]. FMS have been found positively associated with physical activity in 3–6-year-old children [5]. Whereas the longitudinal evidence for a pathway from FMS competence to physical activity level is still indeterminate, individual studies suggest that children who do not adequately develop FMS during childhood are more likely to disengage from organized sports and physical activities later in life [6]. FMS proficiency increases rapidly during early childhood, with a notable acceleration around 45–47 months of age in both boys and girls [7]. However, this progression does not necessarily occur in the same way across sexes. Males tend to demonstrate greater object control skills competence than females, and this difference increases with age [8,9,10].

The debate is still ongoing on which factors, within a comprehensive socio-ecological model of individual, interpersonal, and broader societal determinants of physical activity [11], may be responsible for sex differences in preschoolers’ motor development that are confirmed in large-scale cross-country studies [8]. Given the interrelation between physical activity and motor skill development that emerges as early as preschool age [5], sex differences might also depend on different exposure of females and males to physical activity. In general, opportunities for preschool-aged children to engage in physical activity, FMS, or active play interventions, conducted by trained educators and parents in preschools or childcare centers, positively impact on children’s overall or specific components of motor competence [7]. However, these different types of physical activity seem differently suited to promote motor development at preschool age, with unstructured active play still being reported as less efficacious than structured active play and physical education [12]. The general aim of this study is to explore whether sex differences in unstructured outdoor play might underlie its weaker association with preschoolers’ motor development compared to structured physical activities.

Play is a central component of early childhood development, providing a natural context for exploration, learning, and development. Children are innately drawn to free play, which supports holistic development through self-directed and enjoyable activities [13]. Although free play is not structured to explicitly target cognitive, social–emotional, and motor outcomes, it provides the conditions to facilitate holistic development, particularly in early childhood [14]. Within a wide array of play types, active play seems best suited to promote FMS development [15] as it is “a form of gross motor or total body movement in which young children exert energy in a freely chosen, fun, and unstructured manner” [16].

Throughout the week, young children typically engage in active free play both outdoors and indoors. Outdoor free play (OFP) is defined as unstructured, self-directed play that takes place in outdoor environments [17,18]. It allows children to interact freely with their surroundings and make autonomous decisions about their play. While some activities that children engage in may occur in both indoor and outdoor settings, many physically demanding movements essential for FMS development—such as running, jumping, or kicking—are more commonly and freely expressed outdoors, and not accommodable within indoor spaces such as classrooms or homes.

Nonetheless, in recent years, childhood play patterns have shifted markedly, with daily routines and informal situations in children’s daily lives being dominated by sedentary indoor activities instead of outdoor play and physically active patterns. This trend poses a challenge for parents, teachers, and caregivers, who should ensure that children are provided with stimulating environments that prepare them for dealing effectively with modern world challenges [19]. In this regard, a cross-sectional study conducted with families of children aged 5 to 12 showed that many traditional games, particularly those involving OFP, have disappeared from children’s lives due to erosion of social play contexts [20]. Eymann and colleagues [20] described a generational decline in several outdoor games, while games as tag, hide-and-seek, cops and robbers, and jump rope have persisted, others like ring-a-ring-o’roses and Chinese jump rope are becoming rare. This suggests that within a single generation, children have ceased playing some activities that had been played for at least 2000 years. This ongoing transition has raised international concern.

The UN Committee on the Rights of the Child, General Comment nr. 17 [21], emphasized the importance of OFP as a fundamental right and development opportunity for children to exploit available facilities to be active and to develop their motor skills [22]. Time spent in OFP has been positively associated with greater engagement in moderate-to-vigorous physical activity and reduced time being sedentary [23], both of which are explored as potential predictors of higher FMS proficiency [6].

Despite the great importance attributed to play and specifically OFP by supranational organizations [21], there are several socio-environmental barriers that can prevent children from enjoying their right to play and especially to freely play outdoors. Children’s OFP seems to be favored by the presence of other children nearby to play with and parents’ perception of the benefits of outdoor play, but is hindered by parents who do not allow children to play outdoors without supervision [24]. Since these educational rules are gender-stereotyped [25], it is not surprising that even in OFP contexts, sex-related differences in the activities engaged in by boys and girls may exist, with boys being more likely to engage in OFP than girls [24].

Thus, while the existing literature suggests that OFP positively influences motor skill development in preschool children across sexes, to the best of our knowledge, no studies have examined the role of OFP in the well-documented sex differences in motor competence. Given this context, the current study aims to explore the moderating effect of OFP in the association between sex and FMS in preschool-aged children. Understanding this possible moderation may inform the development of equitable, targeted interventions that support optimal motor development for all children. Considering the higher engagement of boys in OFP compared to girls [24], we hypothesized that boys might exhibit a stronger association between OFP and overall FMS. Also, given that girls are more likely to explore OFP through fine, locomotor, or balance skills [26], we hypothesized that only males might show an association of OFP with object control skills.

## 2. Methods

### 2.1. Ethics

The Ethical Board of the Universities of Verona approved the study (UNVRCLE 34.R2_2021, date of approval 16 March 2023), and the criteria for human research defined in the Declaration of Helsinki [27] were complied with. Preschool principals provided consent, and the parents (or legal guardians) of the participating children gave written informed consent.

### 2.2. Participants and Study’s Protocol

This study is part of the project “Gross motor coordination in boys and girls of preschool age”, which aimed to explore the association between motor competence and OFP in order to contextualize FMS interventions targeted and designed to capitalize on the complementary role of OFP and structured physical activity in the educational context [28]. The study was conducted in 6 conveniently selected kindergartens in urban (*n* = 5) and rural (*n* = 1) areas from Veneto, Italy. All the children aged 3 to 5 years, whose parents or legal guardians consented, were eligible to participate in the study.

Assessments, including anthropometric and FMS measurements, were taken at the preschool gym, during the Physical Education lessons, between 10 a.m. and 11 a.m., from November 2022 to April 2023. All assessments were carried out by trained Physical Education teachers or specifically trained Sport Science students. The presence and collaboration of the curricular PE teachers were guaranteed at any time to instill confidence in the children. The assessor–pupil ratio was 1:2. OFP questionnaires were distributed via children’s backpacks and completed at home by one parent or primary caregiver. The final sample was composed of 142 preschool children (boys: *n* 73; girls: *n* 69), aged between 3 and 5 years.

### 2.3. Anthropometric Assessment

Body height and weight were measured according to a standardized anthropometric measurement protocol [29] and used to characterize the sample. Height was measured with a stadiometer to the nearest 0.5 cm. Weight was measured to the nearest 0.1 kg with an electronic scale, with the subject wearing minimal clothing and without shoes. Body mass index (BMI) was then calculated using height and weight [(weight (kg)/height (m^2^)] and inserted as a covariate in the analyses.

### 2.4. Fundamental Motor Skills Assessment

The Test of Gross Motor Development—3rd edition (TGMD-3)—was used to assess preschoolers’ FMS. It consists of a two-factor test with 13 skills: 6 locomotor skills (run, gallop, hop, skip, horizontal jump, and slide) and 7 ball skills (two-hand strike, one-hand strike, dribble, catch, kick, overhand throw, and underhand throw). Research examining the internal validity of the TGMD-3 showed very high correlations between performance on the locomotor, ball skills, and total TGMD scores (all rs = 0.98) [30].

According to the procedures, children practiced each skill and then had to perform each of the 13 skills twice. For each trial, a child receives a score of “1” if the performance criteria (e.g., stepping with the foot opposite to the throwing arm) are met and a score of “0” if the criteria are not met. The locomotor and ball skills scores are based on the presence (one) or absence (zero) of each performance criterion. For a detailed description of the criteria for each skill, see Ulrich [30]. All skills were video recorded and coded by two experts who had prior experience in coding this assessment. The experts were graduate students from the same department who received standardized training, during which they also independently coded a sample of TGMD-3 behaviors of 3 to 5-year-old children; inconsistencies (inter-observer agreement rate: [Agreements/(Agreements + Disagreements) × 100] > 80%) were solved by consultation. Thereafter, they coded the FMS performances of the children participating in the present study, each receiving a random-stratified half of the children’s videos to code.

### 2.5. Outdoor Free Play Assessment

OFP was assessed using the Children’s Outdoor Play questionnaire [31], validated in Italian by Pesce et al. [28]. The questionnaire includes eight items referring to typical outdoor play locations, administered separately for weekdays and weekend days. Parents were asked to report the number of days during a typical week—without reference to any specific time period—on which their child spent at least 10 min engaging in play at the following locations: (i) the family’s own yard or garden; (ii) a neighbor’s or friend’s yard or garden; (iii) the child’s own street, court, or footpath; (iv) a neighbor’s or friend’s street or footpath; (v) parks or public playgrounds; (vi) sports grounds or outdoor facilities used outside of organized activities; (vii) school sports facilities outside school hours; and (viii) any other outdoor location.

For weekdays, responses were recorded using a five-point Likert-type scale (i.e., 1 = Never or rarely; 2 = Less than once a week; 3 = One or two days per week; 4 = Three or four days per week; 5 = Five days per week). For weekend days, responses were recorded using a six-point scale (i.e., 1 = Never or rarely; 2 = Less than one weekend per month; 3 = One weekend per month; 4 = Two weekends per month; 5 = One day each weekend; 6 = Both days every weekend). Each location was scored independently, and the total OFP score was computed separately for weekdays and weekends as the sum of all item responses.

### 2.6. Statistical Analysis

Descriptive analyses were first conducted to summarize the distributions of all key variables, including sex (n and %), age, BMI, FMS, and OFP (means and standard deviations). Regression analyses were performed using the PROCESS macro for SPSS (version 29.0) (Model 2; ref. [32]) to explore whether the expected prediction of FMS by sex is moderated by OFP. Since both meta-analytic [10] and cross-country research evidence [8], as well as a large-scale study performed in the same country of the present research [33], show nuanced FMS differences between males and females as a function of age within the preschool age span, age was also added to the present analysis model as a potential moderator of sex differences. Thus, sex was treated as the independent variable (X); FMS (i.e., in separate regression analyses, total FMS, locomotor skills, and ball skills raw scores) as the dependent variable (Y); and OFP and age as moderators (M1 and M2). The model was also run accounting for BMI as a covariate. Given the collinearity of OFP at weekends and weekdays, which in regression analyses could inflate the variance of regression parameters and hence potentially lead to bias in the identification of relevant predictors, only one of the two measures of OFP was entered into the regression analyses. According to the validation study of the OFP questionnaire [28], OFP at weekends was considered best suited to represent children’s free outdoor play behavior. The significance of the main and interaction effects (X * M1 and X * M2) was tested using bias-corrected bootstrapping with 5000 resamples. An effect was considered statistically significant if *p* < 0.05 and the 95% confidence interval of the effect coefficient did not include zero.

Then, the interrelationships between FMS (i.e., locomotor and ball skills) and OFP (i.e., weekdays and weekends), accounting for age and BMI were also calculated for both boys and girls, using a “Machine Learning” technique entitled Network Analysis to confirm the linear results through an approach that that evaluates the multiple interactions between variables depicted in graphical representations [34]. Considering the cross-sectional nature of this study, an undirected weighted network analysis was used to estimate the relationship between nodes (variables) from a correlation matrix that, when transformed, is represented by positive or negative edges, which are the relationships between the different nodes. In this sense, to consider the multiple bidirectional interrelationships between the explored variables jointly, four networks, comprising boys and girls, and OFP during week and weekend days, were also created.

The “Fruchterman–Reingold” algorithm was applied so that data were presented in the relative space in which variables with stronger associations remained together, and the less strongly associated variables were repelled from each other [35]. The pairwise Markov random field model was used to improve the accuracy of the partial correlation network, which was estimated from L1-regularized neighborhood regression. The least absolute contraction and selection operator was used to obtain regularization and to make the model less sparse [36]. The Extended Bayesian Information Criterion (EBIC) parameter was adjusted to 0.5 to create a network with greater parsimony and specificity [37].

The qgraph package of the Rstudio (free version) program was used to estimate and visualize the graphs [34]. Regularized algorithms of the selection operator and minimum absolute reduction (LASSO) were used to obtain the precision matrix, which, when standardized, represents the associations between network variables. The thickness and color intensity of the lines represent the magnitude of the associations. The blue lines represent positive associations, and the red lines represent negative ones. The centrality index “Expected Influence”, which indicates the importance of a node for the structure and functioning of the network, was calculated. This centrality index consists of the sum of all possible edge weights that connect one node to another. It was used to assess the nature and strength of a variable’s cumulative influence within the network, and thus, the role it may be expected to play in the activation, persistence, and remission of the network [38]. A positive expected influence means that the influence of that specific node in the network tends to increase, for the acquisition of an adequate network pattern.

## 3. Results

**Figure 1:**
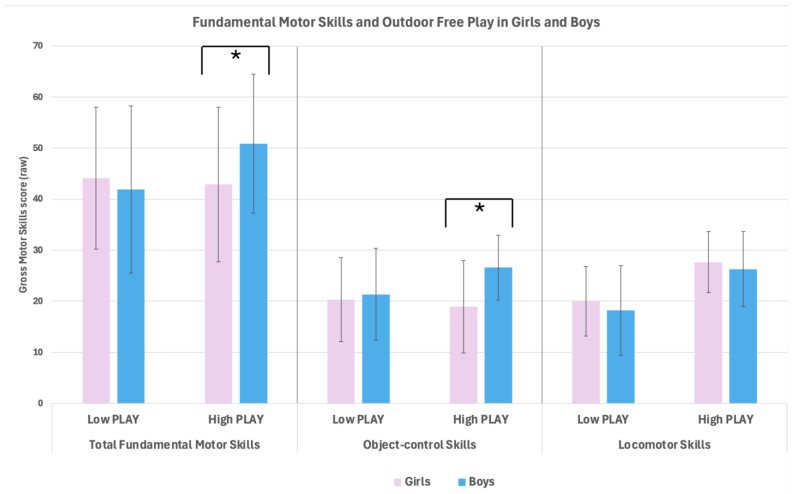
Fundamental motor skills performance as a function of the level of outdoor free play in preschool-aged girls and boys (left: Total; middle: Ball Skills; right: Locomotor Skills). * *p* < 0.001.

**Figure 2:**
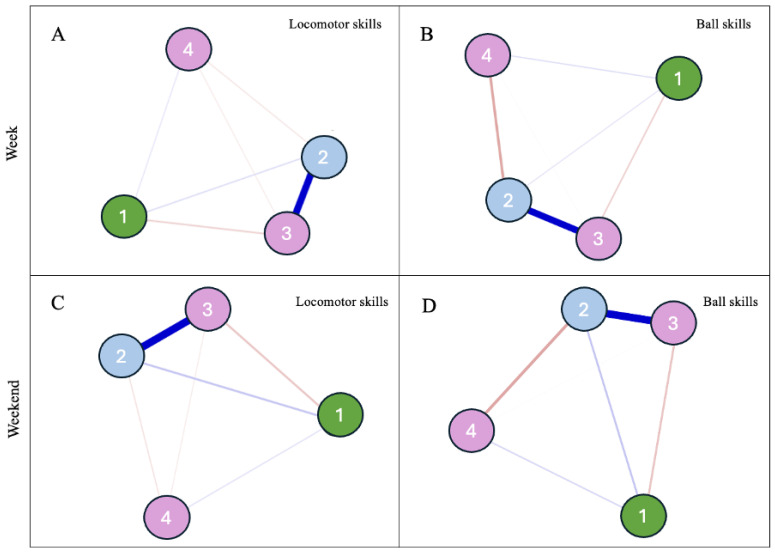
Boys. Relationship between outdoor free play (node 1; (**A**,**B**): play at weekdays; (**C**,**D**): play at weekends), domain of motor skills (node 2; (**A**,**C**): locomotor; (**B**,**D**): ball skills), age in months (node 3), and body mass index (node 4). The blue and red edges express positive and negative relationships, respectively. The thickness of the connecting lines indicates the weight of the ratio.

**Figure 3:**
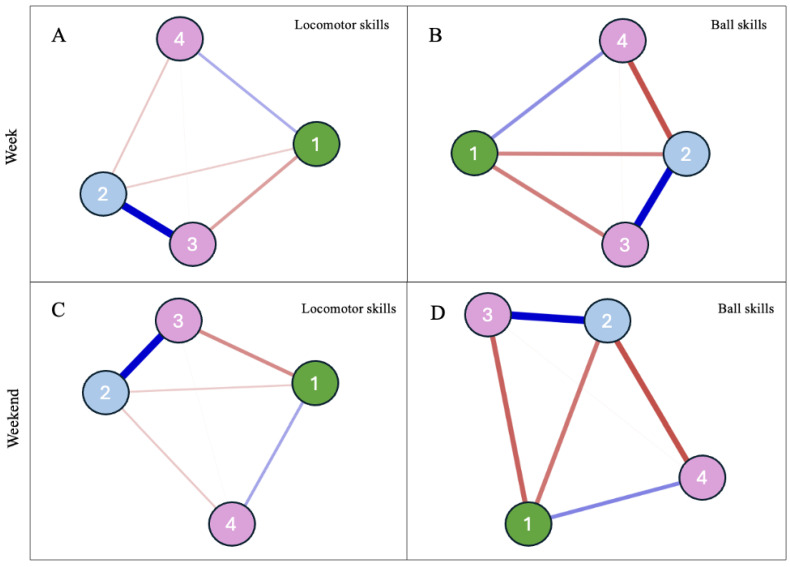
Girls. Relationship between outdoor free play (node 1), domain of moto skills (node 2; (**A**,**B**): play at weekdays; (**C**,**D**): play at weekends), age in months (node 3; (**A**,**C**): locomotor; (**B**,**D**): ball skills), and body mass index (node 4). The blue and red edges express positive and negative relationships, respectively. The thickness of the connecting lines indicates the weight of the ratio.

Descriptive statistics are presented in Table 1 and Table 2 and Figure 1, Figure 2 and Figure 3 show the interaction results that answer the main question of the study on whether and how OFP influences the differences between males and females in FMS (gross motor FMS total: Table 2, left and Figure 1; locomotor skills: Table 2, middle and Figure 2; object control skills: Table 2, right and Figure 3).

The regression analyses explained significant amounts of variance of the raw scores of FMS total (R^2^ = 0.37, F(6,135) = 13.14, *p* < 0.001), locomotor skills (R^2^ = 0.34, F(6,135) = 11.75, *p* < 0.001) and object control skills (R^2^ = 0.32, F(6,135) = 10.72, *p* < 0.001).

For FMS total, the regression revealed a main effect for sex (−28.40, 95% CI [−55.27, −1.52], t = −2.09, *p* = 0.038) and for age (0.67, 95% CI [0.36, 0.98], t = 4.32, *p* < 0.001), no main effect for OFP (*p* = 0.571), but a significant Sex * OFP interaction (0.72, 95% CI [0.15, 1.28], t = 2.50, *p* = 0.013). Similarly, for object control skills, the regression revealed a main effect for sex (−17.27, 95% CI [−33.33, −1.20], t = −2.13, *p* = 0.035) and for age (0.24, 95% CI [0.06, 0.43], t = 2.62, *p* = 0.010), no main effect for OFP (*p* = 0.326), but a significant Sex * OFP interaction (0.40, 95% CI [0.07, 0.74], t = 2.37, *p* = 0.019). Instead, for locomotor skills, there was only a main effect for age (.43, 95% CI [0.26, 0.60], t = 5.00, *p* < 0.001), whereas there was neither a main effect for sex (*p* = 0.146) nor for OFP (*p* = 0.910), and the Sex * OFP interaction did not reach significance (*p* = 0.060).

When considering the complexity of the interrelationships between OFP, FMS, age and BMI in boys and girls via network analysis, the patterns of associations for boys (Figure 2) showed that OFP and age on both week and weekend days were positively related with locomotor and ball skills, whereas BMI was negatively related to both skill types. In Panels A and C, motor skill competence (node 2) and age (node 3) emerged as the variables with the highest expected influence value (1.12 and 0.12; 0.55 and 0.43, respectively), thus meaning that they were the most strongly interconnected with the other variables. Instead, in Panels B and D, BMI (node 4) emerged as the node with the highest expected influence value (−1.13 and −1.18, respectively). The weight matrix is presented as a Supplementary File S1.

For girls, similar to boys, the patterns of associations (Figure 3) showed that OFP and age on both week and weekend days were positively related to locomotor and ball skills, whereas BMI was negatively related to both. Instead, with regard to the nodes’ expected influence value, the pattern of results partially differed from that of boys. Age (node 3) emerged as the variable with the highest expected influence value in Panel A (0.84) and the second highest in Panel C (0.70), where motor skills (node 2) had the highest expected influence value (0.85) in the network of associations. In Panels B and D, age (node 3) emerged as the one with the highest expected influence values (1.40 and 1.30, respectively). Stronger negative associations within the different network patterns were seen for girls when compared to boys. The weight matrix is presented as a Supplementary File S1.

When considering the possible non-linear interrelationships between the assessed variables, the network analysis depicted in Figure 1 and Figure 2 showed different patterns of associations with OFP on weekdays and weekends for boys and girls and reinforced the important role of age and BMI as key associates of FMS, especially of ball skills.

## 4. Discussion

This study contributes to the growing research on the role of children’s play, whose importance for healthy development is increasingly recognized in the public health discourse [39,40]. The aim was to investigate whether differences in FMS between females and males, typically observed in early childhood and beyond, are associated with participation in free outdoor play. This question was addressed cross-sectionally by means of moderated regression analyses. The results confirmed the expected main effects for sex and age, with better FMS performance in boys and with increasing age. Most relevant to the study question, the results also revealed a moderation of sex differences by children’s engagement in free play outdoors. Sex differences were limited to children who frequently engaged in OFP, with boys outperforming girls in overall FMS and specifically object control skills. Instead, sex differences were not found for children who engaged in OFP less frequently. When considering the non-linear interrelationships between the assessed variables, the network analysis depicted different patterns of associations between FMS, OFP, age and BMI; it reinforced a positive association between outdoor play and motor skill competence in males but not in females, and additionally highlighted the role of age and BMI as influential factors of the pattern of differences, between boys and girls, in FMS and especially ball skills.

Our results showed that boys outperformed girls in overall FMS and object control skills but not in locomotor skills, consistent with prior meta-analytic evidence broadly encompassing childhood and adolescence [41] or focusing on early childhood [10]. Also, a large-scale study performed in the same country as the present research [33] consistently showed that preschool boys outperformed age-matched girls in overall FMS and specifically in object control skills, but not in locomotor skills. With the wide-angle lens of cross-country research [8], researchers even found an advantage for preschool girls compared to boys in locomotor skills; however, this advantage was limited to older preschoolers, suggesting a more nuanced pattern of sex differences as a function of age. Our results did not confirm this interactive effect of sex and age on motor skill competence, since older age predicted better motor skill competence in both males and females.

The novelty of our study is the role of outdoor play habits, which seem to moderate the differences between females and males in overall motor skills competence (Figure 1, left) and specifically in object control skills (Figure 1, middle). It is important to note that we did not find a direct association of OFP with motor skill competence, as children who frequently engaged in OFP did not exhibit better FMS performance than their less engaged counterparts. This is at odds with evidence demonstrating, for example, that every additional 10 min of daily outdoor time was associated with an increase in object control in US preschoolers [42]. A Canadian study also demonstrated that children with advanced motor skill competence, compared to their counterparts with emerging motor competence level, played outdoors more frequently [43]. Furthermore, in the European context, there is longitudinal evidence showing that outdoor play time predicted overall FMS and object control skills in preschool children [44], with separate subgroup analyses for boys and girls showing, however, that this prediction was significant only for females.

Instead, in the present study, we found the reverse pattern of higher motor skill competence (overall FMS and object control skills) being associated with more frequent OFP in boys but not in girls. This absence of significant association of higher OFP with higher motor skills competence in girls suggests that their physical activity behavior and motor skills practiced during spontaneous outdoor play may differ from those of their male counterparts. In general, research on young children shows that girls tend to spend less time playing outdoors than their male counterparts [45]. Outdoor play provides opportunities to engage in moderate-to-vigorous physical activity, which, in turn, is associated with motor skill competence at least in preschoolers [46]. Nevertheless, some evidence shows a positive direct association of motor skill competence with outdoor play only for boys, whereas girls’ motor skill competence seems to be rather associated with factors as the school ground aesthetics [43].

Using the lens of a comprehensive socio-ecological model of outdoor play and motor skill competence [43,47], different patterns of OFP in females and males that differently impact their FMS may stem not only from biological factors but also from gendered preferences for different types of play and playgrounds and a different socialization into play [48]. It is worth noting that differences in the association of OFP with motor skill competence in females and males can also depend on how girls and boys spend their time outdoors. This is an ambiguity in most OFP assessments, as playing outdoors is often operationalized and evaluated in terms of time spent outdoors [18]. Although outdoor spaces generally encourage movement [47], OFP is freely chosen by children, and therefore, not all move in the same amounts or in the same ways [49].

On one hand, boys tend to engage more in high-intensity activities that demand strength and motor coordination, are competitive, and predominantly challenging, while girls often participate in quieter, less physically demanding, symbolic or constructive play [48]. On the other hand, access to OFP remains uneven, often shaped by parental perceptions of safety, logistical barriers, and environmental factors, as children with high OFP usually have parents who believe it is safe for their children to play outdoors [50,51]. Indeed, while parents believe that nature play has educational value, regulates children’s emotions, and helps discover their abilities, the time required to accompany them, as well as weather conditions, act as barriers to nature play engagement [45,52]. These challenges may differentially impact girls, particularly if their play opportunities are less physically active or more constrained by adult supervision or cultural expectations. Indeed, gender normative parental support seems to be a contributing factor to the observed difference in motor skill competence between females and males [41] and may also contribute to the differential association with OFP observed in the present study. The culture of equal opportunities for females and males that is more strongly rooted in North European countries might explain why, in contrast to our findings, a study performed with preschoolers in Finland could demonstrate that girls, especially, could capitalize on OFP opportunities to develop their FMS [44].

While sports and structured physical activities are common for older children, OFP is the primary form of physical activity for children at an early age. Within the preschool period, it is relevant to reinforce parents, teachers, and caregivers’ key role in promoting equitable OFP opportunities and exposing children to enriched physical activities, emphasizing the need for targeted strategies to support FMS development. Environmental enrichment includes structured playgrounds, diverse materials, or adult facilitation to broaden the types of play [53,54]. Moreover, the complementariness of OFP in early childhood and environmental enrichment tailored to foster FMS development emerges from studies, in which enrichment is generated by means of structured physical activities [28,44]. OFP seems to contribute, along with structured multisport and physical activity games, to the development of motor skill competence, acting as an independent predictor [44] or as a moderator that amplifies the effects of structured and deliberate play [28].

The current results also confirmed the well-established association between age and FMS, showing that children’s FMS proficiency increases throughout early childhood. This aligns with extensive evidence demonstrating that FMS develop progressively from ages 3 to 5 and continue improving throughout the school years [7,11,33,55]. FMS proficiency evolves at different rates across skill domains, with locomotor skills typically emerging earlier and showing less sex disparity than object control skills, which favor boys even at young ages [33,44,56].

Although the abovementioned associations among sex, age, play habits, and motor skill competence have been commonly examined separately, they influence each other mutually. Thus, it makes sense to examine all assessed variables together (Figure 2 and Figure 3). This study adds important information to the literature, accounting for the non-linear, dynamic, and complex relationship between sex, FMS, and OFP. The exploratory network analysis confirmed a positive association between OFP and motor skill competence in males but not in females. Moreover, it evaluated the interrelatedness of sex, FMS, and OFP with other variables that had been entered as covariates (BMI) or parallel moderators (age) in the moderated regression model focused on the contribution of OFP to sex differences in FMS. Within this pattern of non-linear associations, age and BMI emerged as critical variables to intervene in boys and girls in order to achieve a positive systemic profile (i.e., variables with the highest expected influence value). Although the results have highlighted locomotor skills as a node with high expected influence value, highlighting its central role in the network of associations especially in the case of males, the results also reinforce how changes in age and BMI may ripple through the entire system, due to their overall influence in shaping the structure and dynamics of the network. This result is in line, for example, with systematic reviews on the inverse relationship between BMI and FMS [6]. Also, the higher negative interrelations depicted in the networks for girls reinforce the need for special attention in this critical subgroup of the population, especially when considering ball skills.

This study has limitations that need to be addressed. First, the cross-sectional nature of the data does not allow for the inference of causality. It can only be speculated that different ways females and males spend their time outdoors, with males engaging to a larger extent in free gross-motor play and ball games, may be responsible for the higher motor skill competence observed only in males who engage in higher levels of OFP. Second, even though a validated instrument was used, OFP was assessed by means of a parent-reported questionnaire, which has an associated recall or social desirability bias. Future studies could further explore this association using objective tools to confirm the duration or intensity of OFP. The lack of information about the nature of the activities during OFP, as well as the environmental conditions, is a limitation that could be further explored. Finally, the relatively small size of the convenience sample limits the generalizability of the results but provides effect sizes that are useful for estimating the appropriate sample size for future longitudinal studies with a moderated prediction analysis model. However, the network approach used was sufficiently robust to mitigate the possible impact of the sample size, and its results reinforced the results observed in the moderated prediction analysis.

## 5. Conclusions

The results suggest that, as early as preschool age, males may capitalize better than females on free outdoor play opportunities to engage in gross-motor activities with a ball that promote their motor and especially object control skill development. Future longitudinal studies are needed to test this speculative causal hypothesis. Relevant implications regard encouraging OFP as part of preschoolers’ routines to support their FMS development and fostering female children’s interest in gross-motor ball games to enable them to exploit the opportunities provided by free play in outdoor environments to develop the full range of FMS competence.

## Figures and Tables

**Table 1:** Descriptive statistics of the sample.

		Males (*n* = 73)	Females (*n* = 69)	Whole Sample (*n* = 142)
Age (months)	mean	52.29	51.15	51.73
	(SD)	(9.74)	(9.88)	(9.7)
Body Mass Index	mean	15.76	15.96	15.86
	(SD)	(1.28)	(1.74)	(1.52)
Outdoor Free Play—Weekend	mean	23.92	22.61	23.28
	(SD)	(7.11)	(7.75)	(7.43)
Outdoor Free Play—Weekdays	mean	17.52	17.01	17.27
	(SD)	(4.78)	(5.19)	(4.97)
TGMD total FMS	mean	47.05	43.44	45.30
	(SD)	(15.21)	(14.95)	(15.14)
TGMD Locomotor Skills	mean	22.85	23.85	23.33
	(SD)	(7.48)	(8.91)	(8.19)
TGMD Object Control Skills	mean	24.34	19.59	22.03
	(SD)	(9.05)	(7.74)	(8.74)

**Table 2:** Gross motor, locomotor, and ball skills (raw scores) in girls and boys by level of Outdoor Free Play at the weekend (dichotomized at median in lower and higher outdoor free play—OFP level).

	Total Fundamental Movement Skills (Raw Score)		Locomotor Skills (Raw Score)		Ball Skills (Raw Score)	
	Girls	Boys	Girls	Boys	Girls	Boys
Lower OFP						
(Mean, SD)	44.06 ± 16.36	41.90 ± 13.93	19.97 ± 8.78	18.19 ± 6.79	20.29 ± 8.99	21.32 ± 8.25
Higher OFP						
(Mean, SD)	42.86 ± 13.66	50.86 ± 15.15	27.63 ± 7.38	26.29 ± 6.00	18.91 ± 6.35	26.57 ± 9.06

## Data Availability

Data supporting reported results will be publicly archived, and the link to the repository will be made available once the paper is published.

## Supplementary Materials

The following supporting information can be downloaded at https://www.mdpi.com/article/10.3390/children12050594/s1; Supplementary File S1: Informed consent and Supplementary results of network analysis.

## Abbreviations

The following abbreviations are used in this manuscript:

OFP	Outdoor free play
FMS	Fundamental motor/movement skills
BMI	Body mass index
