# Supplementary material for: Motor Skill Development at Preschool Age in Girls and Boys: The Role of Outdoor Free Play
Source: Children (Basel). 2025 May 2;12(5):594. doi: 10.3390/children12050594 (PMC12109917; doi:10.3390/children12050594)
Supplement: Supplementary file 1 [file children-12-00594-s001.zip › children-3590853-supplementary.pdf]

Supplementary file – S1:

- Additional statistics of the network analysis

- Informed consent

Boys – ball skills - weekdays

Weights matrix

Variable	Network			
	Age_month	BMI	BALL	Play_week
Age_month	0.000	−0.011	0.449	−0.084
BMI	−0.011	0.000	−0.158	0.059
BALL	0.449	−0.158	0.000	0.049
Play_week	−0.084	0.059	0.049	0.000

Boys – ball skills weekends

Weights matrix

Variable	Network			
	Age_month	BMI	BALL	Play_weekend
Age_month	0.000	−0.009	0.453	−0.106
BMI	−0.009	0.000	−0.162	0.070
BALL	0.453	−0.162	0.000	0.101
Play_weekend	−0.106	0.070	0.101	0.000

Boys – locomotor skills week days

Weights matrix

Variable	Network			
	Age_month	BMI	LOC	Play_week
Age_month	0.000	−0.046	0.544	−0.099
BMI	−0.046	0.000	−0.059	0.057
LOC	0.544	−0.059	0.000	0.076
Play_week	−0.099	0.057	0.076	0.000

Boys – locomotor skills weekends

Weights matrix

Variable	Network			
	Age_month	BMI	LOC	Play_weekend
Age_month	0.000	−0.044	0.547	−0.124
BMI	−0.044	0.000	−0.062	0.061
LOC	0.547	−0.062	0.000	0.122
Play_weekend	−0.124	0.061	0.122	0.000

Girls – ball skills weekdays

Weights matrix

Variable	Network			
	Age_month	BMI	BALL	Play_week
Age_month	0.000	−0.013	0.350	−0.186
BMI	−0.013	0.000	−0.254	0.157
BALL	0.350	−0.254	0.000	−0.175
Play_week	−0.186	0.157	−0.175	0.000

Girls – ball skills – weekends

Weights matrix

Variable	Network			
	Age_month	BMI	BALL	Play_weekend
Age_month	0.000	−0.013	0.350	−0.228
BMI	−0.013	0.000	−0.254	0.171
BALL	0.350	−0.254	0.000	−0.200
Play_weekend	−0.228	0.171	−0.200	0.000

Girls – locomotor skills weekdays

Weights matrix

Variable	Network			
	Age_month	BMI	LOC	Play_week
Age_month	0.000	−0.013	0.480	−0.186
BMI	−0.013	0.000	−0.110	0.157
LOC	0.480	−0.110	0.000	−0.100
Play_week	−0.186	0.157	−0.100	0.000

Girls – locomotor skills weekends

Weights matrix

Variable	Network			
	Age_month	BMI	LOC	Play_weekend
Age_month	0.000	−0.013	0.480	−0.228
BMI	−0.013	0.000	−0.110	0.171
LOC	0.480	−0.110	0.000	−0.102
Play_weekend	−0.228	0.171	−0.102	0.000

MODULO DI CONSENSO INFORMATO

RAZIONALE DELLO STUDIO

Gentili famiglie, con la presente la sottoscritta prof.ssa Valentina Biino in collaborazione con l'Università degli Studi di Verona propone lo studio di ricerca dal titolo "CoordinataMente", in merito allo sviluppo delle capacità coordinative in età evolutiva, con lo scopo di valutare la coordinazione motoria nei bambini in età compresa tra 3-6 anni. La proposta del protocollo sperimentale di ricerca è stata rivolta al comitato etico.
A questo proposito, in riferimento agli articoli 13 e 14 del regolamento n. 2016/679 (UE) del Parlamento Europeo e del Consiglio del 27 Aprile 2016 relativo alla protezione delle persone fisiche con riguardo al trattamento dei dati personali, nonché alla libera circolazione di tali dati, fornisce all'interessato le seguenti informazioni.

INFORMATIVA RIGUARDO AL TRATTAMENTO DEI DATI RACCOLTI:

1. I dati saranno tenuti dalla prof.ssa Valentina Biino e-mail: vale.biino@gmail.com. Il responsabile della protezione dei dati è la professoressa stessa.

    - Le finalità del trattamento cui sono destinati i dati sono esclusivamente per valutare l'andamento della coordinazione motoria dei bambini e delle bambine in età compresa tra 3-6 anni)
    - I dati personali raccolti saranno destinati anche ai membri dello staff di ricerca: prof/dott Luciano Bertinato e dott. Matteo Giuriato (Università degli Studi di Verona).
    - Il titolare del trattamento NON ha intenzione di trasferire i dati a un paese terzo o ad una organizzazione internazionale. Per ottenere una copia di tali dati in formato digitale si può fare richiesta all'indirizzo del responsabile della protezione dei dati che sarà tenuto a metterli a disposizione in formato digitale entro 20 giorni lavorativi dalla richiesta.

2. I dati con l'identificazione dei soggetti verranno conservati per diciotto mesi. Successivamente verranno conservati eliminando qualsiasi corrispondenza tra i codici di identificazione i soggetti reali. L'interessato ha il diritto di chiedere al titolare del trattamento l'accesso ai dati personali, la rettifica, la cancellazione degli stessi o la limitazione del trattamento, oppure la possibilità di opporsi al loro trattamento. L'interessato può avvalersi del diritto di revocare il consenso in qualsiasi momento e/o proporre reclamo ad un'autorità di controllo. Ad ogni modo l'interessato NON è obbligato a fornire i dati personali e NON esiste alcuna conseguenza nel caso in cui non le fornisca. La fonte da cui hanno origine i dati personali è unicamente quella fornita dall'interessato e non provengono da fonti accessibili al pubblico. Fornendo i dati personali, l'interessato contribuisce a costituire un campione di studio di una popolazione a cui il progetto di ricerca è interessato.

3. il titolare del trattamento dei dati si impegna a fornire tali informazioni entro un tempo ragionevole dall'ottenimento dei dati personali, ma al più tardi entro un mese. Nel caso in cui siano destinati alla comunicazione con il destinatario o ad altro destinatario, non più tardi della prima comunicazione.

4. Il titolare del trattamento, nel caso in cui intenda trattare ulteriormente i dati personali per una finalità diversa da quella richiesta inizialmente, prima di tale ulteriore trattamento, si impegna a fornire all'interessato informazioni in merito a tale diversa finalità e tutto quanto sopra riportato.

DESCRIZIONE DEL PROTOCOLLO

In accordo con le/gli insegnanti curricolari, durante l'orario scolastico, i componenti dello staff di valutazione proporranno le seguenti misure e condurranno le prove dei seguenti test:

PARAMETRI ANTROPOMETRICI

- Altezza
- Massa

COORDINAZIONE GROSSO-MOTORIA

TGMD-3 (Ulrich D.A., 2019). Test coordinativo che misura il processo di coordinazione grosso motoria in bambini di età tra 3-0 (3 anni e 0 mesi) a 10-11 (10 anni e 11 mesi)

È composto da : Locomotor subtest

Ball skills subtest

Locomotor Subtests. Questo test misura le seguenti capacità grosso motorie che richiedono fluidità, movimenti coordinati del corpo quando il bambino si muove da una direzione ad un'altra.

- Corsa: l'abilità di avanzare costantemente con balzi molleggiati in modo che entrambi i piedi si allontanino dal terreno per un istante con ogni passo (fase di volo)
- Galoppo: l'abilità di svolgere l'andatura a 3 tempi velocemente e naturalmente
- Salto: l'abilità di saltare una minima distanza con il piede preferito
- Skip: l'abilità di skippare in continuazione ritmicamente, alternando i passi
- Salto orizzontale: l'abilità di riuscire ad eseguire un salto orizzontale da in piedi da fermo
- Slide (avanzare): l'abilità di avanzare lateralmente lungo una linea dritta da un punto ad un altro.

Ball Skill subtest. Questo test misura le seguenti capacità grosso motorie che dimostrano efficaci movimenti di lancio, mira e presa

- Colpire con 2 mani una palla ferma: l'abilità di colpire una palla ferma con una mazza di plastica
- Colpo di dritto con una mano una palla a rimbalzo: l'abilità di colpire una palla a rimbalzo con una racchetta di plastica
- Palleggio fermo ad una mano: l'abilità di palleggiare una palla da basket un minimo di. 4 volte con la mano preferita prima di afferrare la palla con entrambe la mani senza muovere i piedi
- Presa a 2 mani: l'abilità di prendere una palla che è stata lanciata sottomano (dal basso)
- Calciare una palla ferma: l'abilità di calciare una palla ferma con il piede preferito
- Lancio overhand (da sopra): l'abilità di lanciare la palla in un punto del muro con la mano preferita
- Lancio underhand (da sotto): l'abilità di lanciare la palla in un punto del muro con la mano preferita

CONSENSO

Con il presente modulo il/la sottoscritto/a____

acconsente alla partecipazione del figlio/a_____________________________________ allo studio

dichiaro di essere stato informato dallo staff di ricerca della natura dello studio e delle sue finalità, nonché delle modalità di esecuzione, degli eventuali rischi e delle analisi che saranno seguite. Le informazioni ricevute sono state chiare ed esaurienti.
Acconsento al trattamento dei dati personali e di quelli derivati dallo studio, per i soli scopi di ricerca.

Verona, ……………………..

Firma del genitore o di chi ne fa le veci Il responsabile della ricerca
